# Supplementary material for: Cold Shock Proteins Mediate Transcription of Ribosomal RNA in Escherichia coli Under Cold-Stress Conditions
Source: Biomolecules. 2025 Sep 29;15(10):1387. doi: 10.3390/biom15101387 (PMC12562117; doi:10.3390/biom15101387)
Supplement: Supplementary file 1 [file biomolecules-15-01387-s001.zip › biomolecules-3793079-supplementary-1.1.pdf]

Supplemental Data

Table S1. Primer sequences.

Gene	Primers	Primer sequences（5′—3′)
pETM-11	Vt-F	CTAGTGGTACCGGATCCGAATTCG
	Vt-R	GCCCTGAAAATAAAGATTCTCAGTAGTGG
cspA	cspA-F	CTTTATTTTCAGGGCATGTCCGGTAAAATGACTGGTATCG
	cspA-R	ATCCGGTACCACTAGTTACAGGCTGGTTACGTTACC
cspB	cspB-F	CTTTATTTTCAGGGCATGTCAAATAAAATGACTGGTTTAG-TAAAATGGTTTAACG
	cspB-R	GATCCGGTACCACTAGTTAATCAGTAATGATGACATTT-GCTGCTGC
cspE	cspC-F	CTTTATTTTCAGGGCATGTCTAAGATTAAAGGTAAC-GTTAAGTGGTTTAATGAG
	cspC-R	GATCCGGTACCACTAGTTACAGAGCGATTACGTTTGCAGC
cspG	cspG-F	CTTTATTTTCAGGGCATGTCTAATAAAATGACTGGTTTAG-TAAAATGG
	cspG-R	GATCCGGTACCACTAGTTAGAGCGTAACAACGTTCGCTG
cspI	cspI-F	CTTTATTTTCAGGGCATGTCTAACAAAATGACTGG
	cspI-R	GATCCGGTACCACTAGTCAAAGCGCCACTACATG
Verify Pri-mer	T7-F	GCTAGTTATTGCTCAGCGGTGG
	T7-R	CCCGCGAAATTAATACGACTCACTATAGG
rRNA Template	pkk3535-F	GTATGGCAATGACGCCAGGAGCTG
	pkk3535-R	CATTGAGTAATTGTTGTTCGTCTTCGGCACATAC
EMSA test promoter fragment	pkk3535-F	GTATGGCAATGACGCCAGGAGCTG
	pKKprm-R	CGTTCAATCTGAGCCATGATCAAAC
EMSA test rDNA frag-ment	pKK450-F	GAAGAGTTTGATCATGGCTCAGATTGAAC
	pKK450-R	CCCCGCTGAAAGTACTTTACAACC
Molecular beacon 1		6-FAM-CCGCGCTCTGAGCCATGATCAAACTCTTCAATTT-GCGCGG-Dabcyl
Complementary oligonu-cleotide 1		AAATTGAAGAGTTTGATCATGGCTCAGA
Molecular beacon 2		6-FAM-CCGCGCATCTCGGTTGATTTCTTTTCCTCGGGCGCGG-Dabcyl
Complementary oligonu-cleotide 2		CCGAGGAAAAGAAATCAACCGAGAT
Molecular beacon 3		6-FAM-CCGCGCGGTTAAGCCTCACGGTTCATTAGTAGCGCGG-Dabcyl
Complementary oligonu-cleotide 3		TACTAATGAACCGTGAGGCTTAACC

 https://doi.org/10.3390/xxxxx

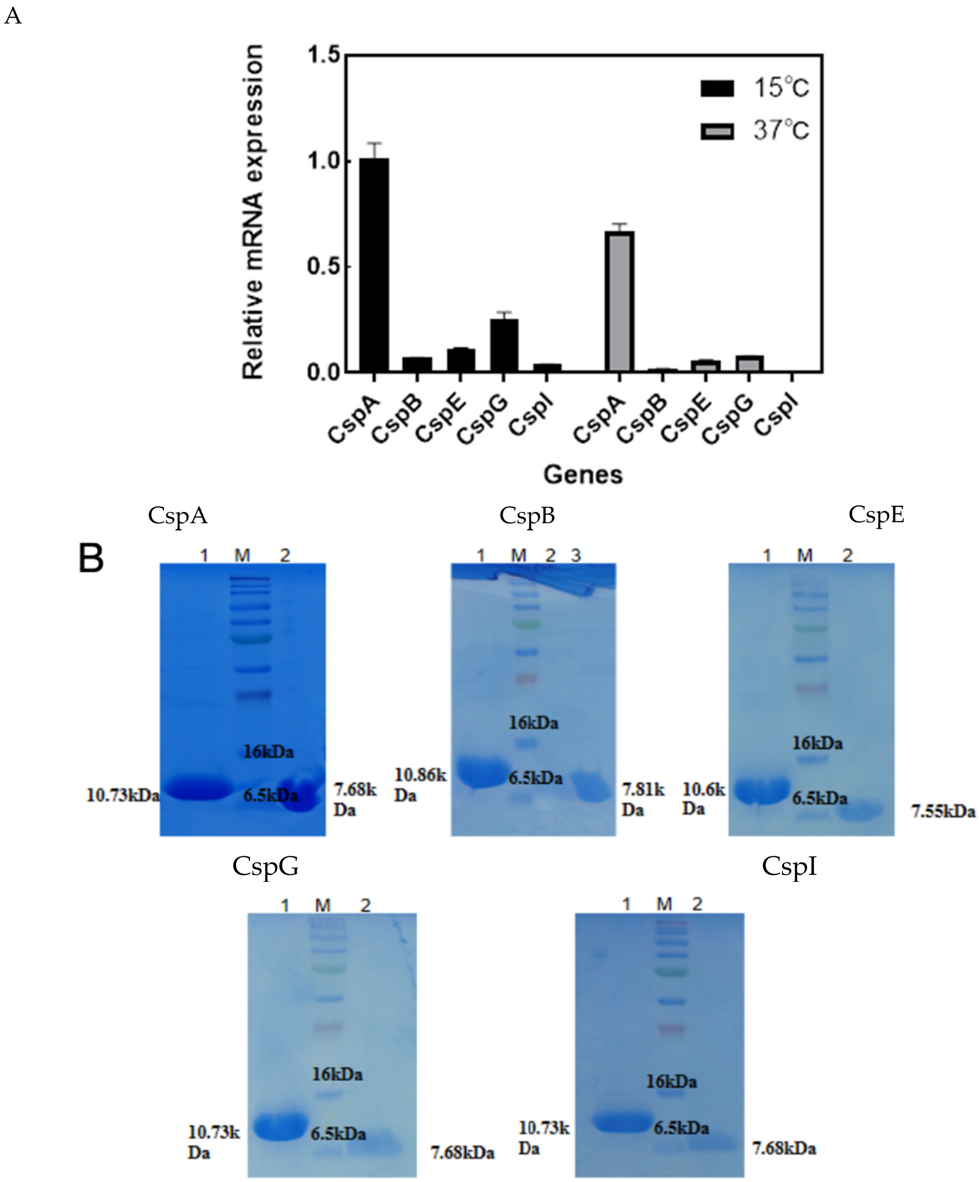

Figure S1. A) Expression levels at 37℃ and 15℃ of five mRNAs encoding the indicated cold shock proteins. B) Electrophoretic analysis of five cold shock proteins after expression and purification. Which M indicate 6.5~270kDa protein marker, Left side of each M are CSPs protein with 6XHis-tag. Right side of M are CSPs protein without 6XHis-tag, that degested by TEV enzyme. (original images can be found in the supplementary material).

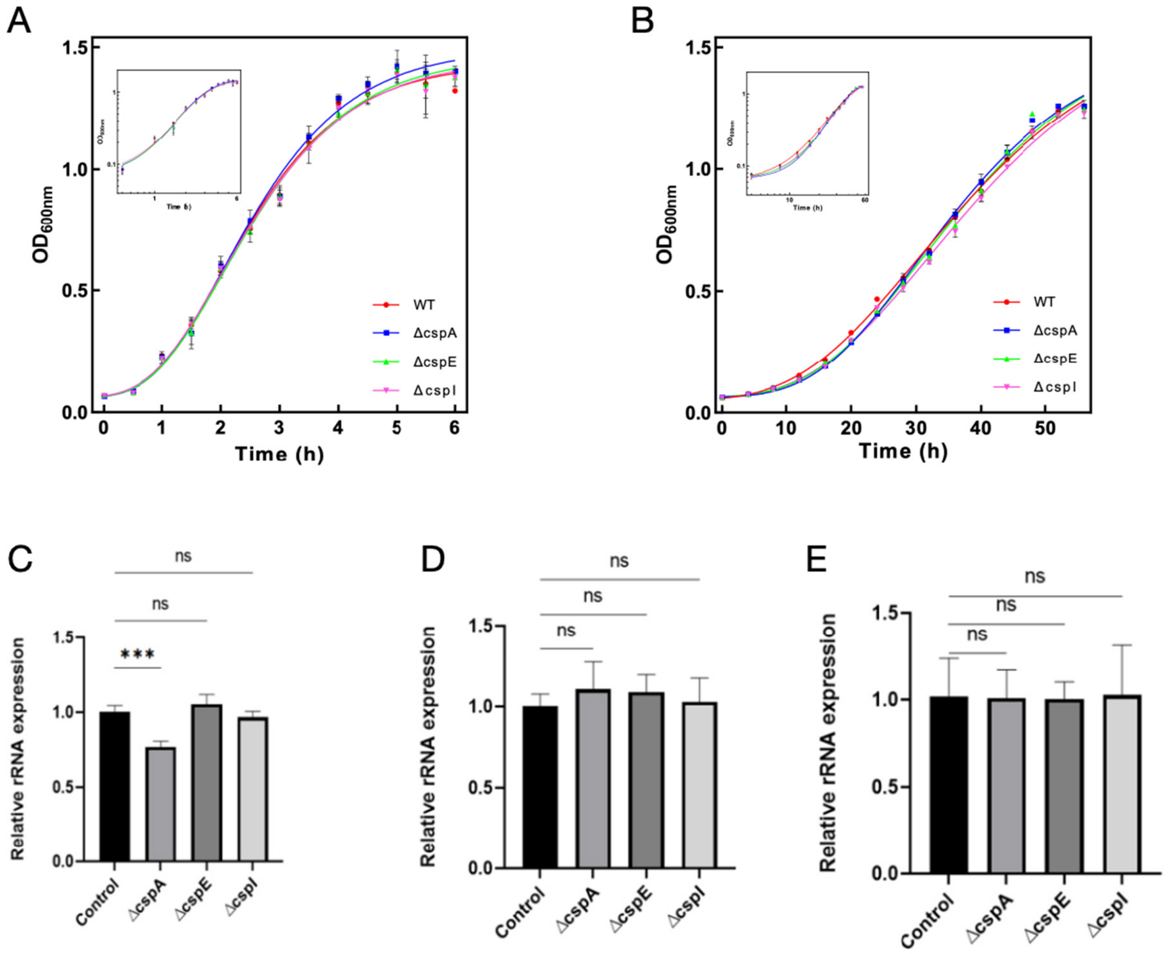

Figure S2. Growth curves and levels of 16S rRNA in single gene deletion strains. Growth curves of single *csp* deletion mutants and wild-type at 37℃, (A) and 15℃ (B). The fitting of the experimental points was carried out using the Gompertz curve equation as described in Materials and Methods. The insets show the log vs log plots. Error bars indicate the standard deviation calculated from three independent experiments. Quantification of 16S rRNA was carried out by real time PCR on total RNA extracted from wt and from the indicated double gene deletion mutants grown at 37°C (C) or 15°C (D, E) up to OD_{600} = 0.3 (C, D) OD_{600} = 0.5 (E). The experimental conditions are described in Materials and Methods. The relative expression refers to the ratio between the amount of 16S rRNA in each strain and that of the wild type strain, measured using the $2^{-\Delta\Delta Ct}$ method. Error bars indicate the standard deviation calculated from triplicate measurements. Data were analyzed using the Anova test. (***: p<0.001; ns: p>0.05).

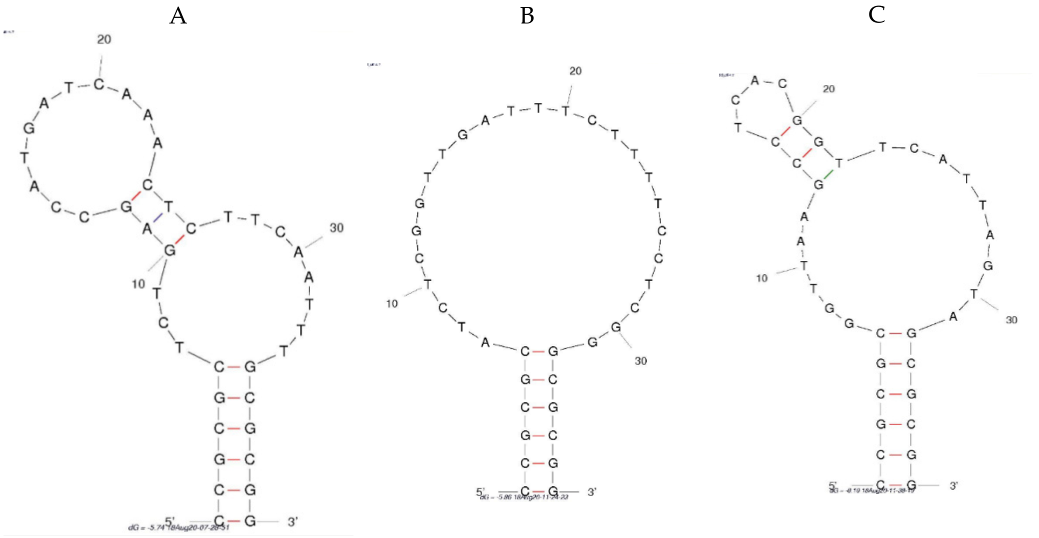

Figure S3. Molecular Beacon sequence and fold scheme. Molecular Beacon 1(M.B.1) (A), M.B.2 (B),M.B.3(C) using the mFold program. Molecular beacons and oligonucleotides show in Table S1were purchased from ComateBio, Changchun. The 5′ end of the molecular beacon is linked to a fluorescent dye (6-FAM), while the 3′–end is coupled to a quencher molecule (Dabcyl). The region which base-pairs with the target (oligo or RNA) is indicated in color.

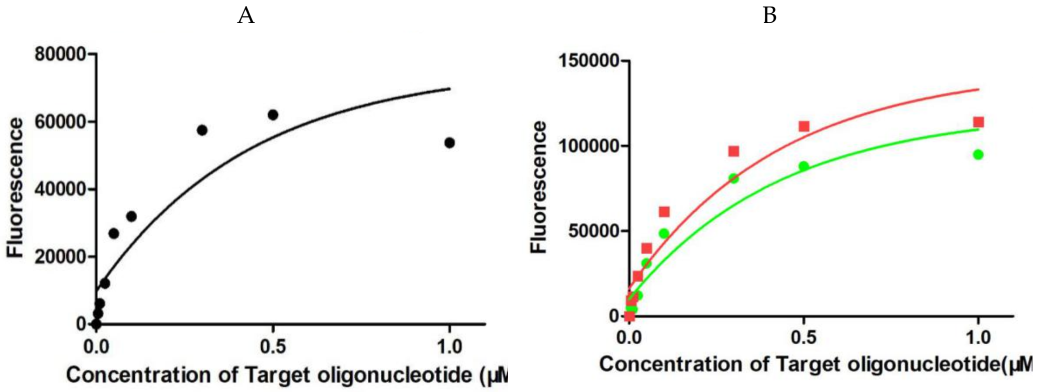

Figure S4. Fluorescence emitted by M.B.1 (A) M.B.2 (B. green) and M.B.3 (B. red) after incubation with the indicated concentration of complementary target oligonucleotides. To establish the range of concentrations of the target required for obtaining a proportional liner fluorescent emission, fluorescence was measured after mixing the M.B. with increasing oligo target concentrations. The result of these experiments, shown in Fig. S8, indicates that the fluorescent signal is linearly related to the concentration of the target when this latter is < then the concentration of the M.B. Furthermore, the emission reaches a plateau when the target is present in about a 3-fold molar excess with respect to the M.B.

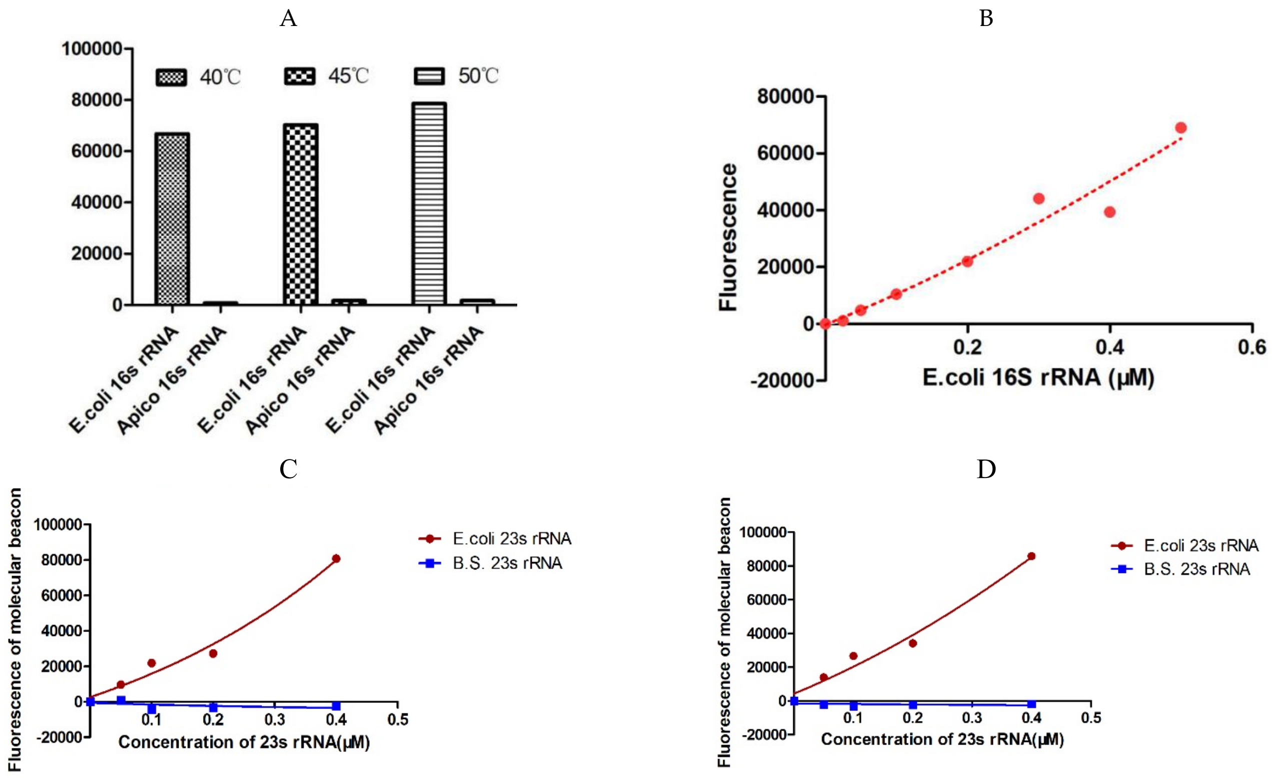

Figure S5. Molecular beacon's specificity and sensitivity test. Fluorescence emitted by 50 nM M.B.1 after incubation with 300 nM of the indicated 16S rRNA as a function of temperature (A). Signal emitted by M.B.1 after incubation with the indicated concentrations of *E. coli* 16S rRNA(B). Fluorescence emitted by M.B.3 (C) and M.B.5 (D) after incubation with the indicated concentration of *E. coli* 23S rRNA (red) or *G. stearothermophilus* 23S rRNA (blue). To demonstrate that the addition of the rRNA produced the expected fluorescent signal. To this aim, we purified the 16S rRNA from the *E. coli* 30S subunits. As a control, in the experiment we also used the in vitro transcribed 16S rRNA of *Plasmodium berghei apicoplast.* In fact, *Plasmodium* species carry two organelles: the *mitochondrion* and the *apicoplast*. The apicoplast is a non-photosynthetic plastid of algal origin, a product of secondary endosymbiosis. The *apicoplast* organelle contains a DNA molecule of about 35 kb which encodes for less than 50 proteins, many of which involved in the organelle's transcription and translation processes as well as for the related *apicolast* ribosomal RNAs (SSU and LSU rRNA genes) and transfer RNAs (tRNA clusters). In line with the evolutive history of the *apicoplast*, the genes encoded in the *apicoplast* genome display an evident bacterial origin, as demonstrated by phylogenetic analysis. However, despite the bacterial origin, the *apicoplast* 16S rRNA is completely different in the regions bound by the M.B. in terms of nucleotide sequence. To demonstrate that the addition of the rRNA produced the expected fluorescent signal. To this aim, This latter rRNA was used as a control, being rather differ in the regions bound by the M.B.s in terms of nucleotide sequence. were then mixed in increasing amounts with 50 nM of the M.B.s in separate reactions. The result of these experiments, shown in Fig 6. C/D , indicate that the M.B.2/3 bind specifically the target *E. coli* 23S rRNA producing a good fluorescent signal which increases with increasing rRNA amounts. The minimum amount of rRNA which can be detected in this system is 0.5-1 pMol which corresponds to 0.5-1 µg of full length 23S rRNA. Specifically, the target *E. coli* 16S rRNA producing a good fluorescent signal in the range of tested temperatures. Subsequently, we also tested the response of the M.B. to increasing amounts of *E. coli* 16S rRNA (Fig. 6B). The result shows that fluorescence increases with increasing rRNA amounts. The minimum amount of rRNA which can be detected in this system is 0.5-1 pmole which corresponds to 0.25-0.5 µg of full length 16S rRNA.
